# Transient Neuronal Injury Followed by Intravascular Injection During an Ultrasound Guided Stellate Ganglion Block

**DOI:** 10.5812/aapm.7823

**Published:** 2013-01-01

**Authors:** Hariharan Shankar, Swetha Simhan

**Affiliations:** 1 Department of Anesthesiology, Clement Zablocki VA Medical Center, Medical College of Wisconsin, Milwaukee, USA; 2 Department of Anesthesiology, Medical College of Wisconsin, Milwaukee, USA

**Keywords:** Ultrasonography, Complications, Stellate Ganglion

## Abstract

Ultrasound guidance for pain interventions is becoming increasing recognized as a useful imaging tool. One of the common interventions where it is gaining wider acceptance is during the performance of a stellate ganglion block. The following is a unique report where intravascular and neuronal injury occurred during the performance of an ultrasound guided stellate ganglion block followed by dysphagia. 41 year old male, with a diagnosis of complex regional pain syndrome, was referred to our clinic for further management. He underwent a diagnostic ultrasound guided stellate ganglion block after having tried conservative therapies. The stellate ganglion block provided him with complete pain relief for over five weeks. During a subsequent therapeutic stellate ganglion block, performed by an experienced pain medicine fellow with more than 50 ultrasound guided proceduresclinician, the patient developed a transient injury to the brachial plexus upon needle entry. Subsequent redirection and injection of an ml of injectate resulted in an intravascular injection producing tinnitus. After the tinnitus decreased, he underwent another stellate block using an out of plane approach without any further complications. Two days later, he reported chest and throat discomfort which resolved over the next few days possibly due to a retropharyngeal hematoma. He declined further interventions and was subsequently managed with 3 tablets of oxycodone a day. This report highlights the importance of vigilance and meticulous planning during the performance of ultrasound guided pain interventions.

## 1. Introduction

Diagnostic and therapeutic paravertebral sympathetic ganglia blocks are utilized for a variety of sympathetically maintained painful conditions. Reports of retropharyngeal hematoma, hypertension, osteomyelitis of the cervical spine, locked in syndrome, subdural and intravascular injections following stellate ganglion blocks, despite being rare, prompted the quest for safer image guided techniques (1-7). The Last decade has seen a trend away from landmark based to image guided injection techniques for the performance of these blocks. Compared to fluoroscopy and CT, ultrasound imaging lends itself as an ideal tool for stellate ganglion block, because of its clarity, portability, low cost and lack of radiation. High resolution sonographic imaging of the neck provides visualization of osseous as well as soft tissue anatomy including vasculature, thyroid gland, and oesophagus (8). Inadvertent intravascular injections or neuronal injuries have been reported with ultrasound guided regional anesthesia (9, 10). Procedural success and risk reduction during ultrasound guided interventions are determined by familiarity with anatomy, technical skills, and needle trajectory planning during a scout scan (11). Cardiac arrest, secondary to intravascular injection, has been reported following ultrasound guided stellate ganglion blocks (12). This brings to question the infallibility of ultrasound guidance, promotes retrospection and an understanding of its limitations. The following is a unique report where a transient injury to the brachial plexus was followed by an intravascular injection with symptoms of central nervous system local anesthetic toxicity during the performance of ultrasound guided stellate ganglion block.

## 2. Case Report

**Figure 1.**
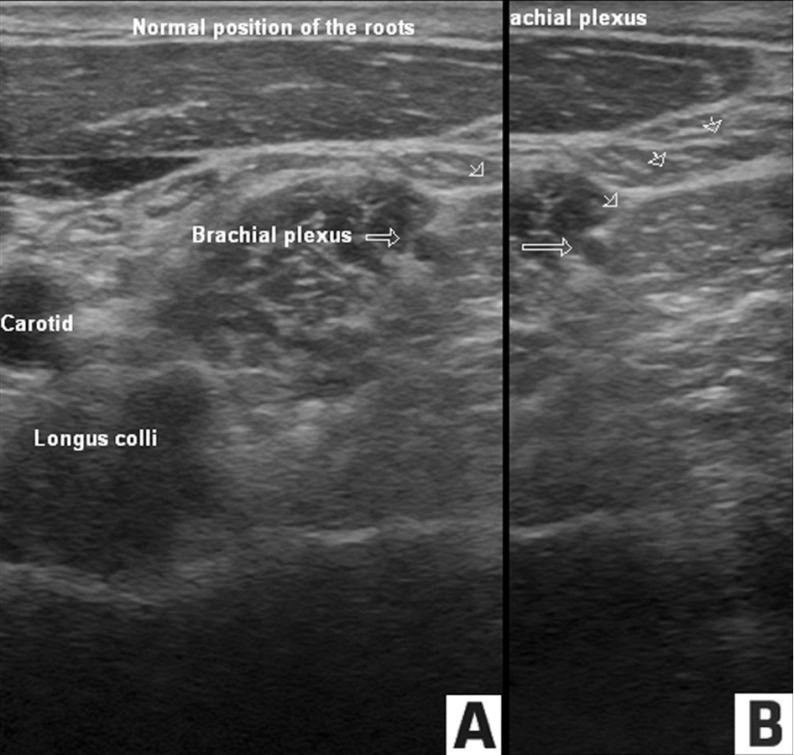
Ultrasound images over the anterior neck at the level of the brachial plexus roots. A) Normal position of the brachial plexus roots when a 25G needle (arrowheads) was just introduced above the brachial plexus roots (arrow). B) The fascial sheath enclosing the brachial plexus roots (arrow) is indented producing a lengthening of the distance, compared to figure 1A, when the needle (arrowheads) was advanced further. This also resulted in paresthesia.

A 41 year old male was referred to us in 2008 with a diagnosis of Complex regional pain syndrome (CRPS) of bilateral wrists, following an electric current burn during welding in 2003. He described the pain as sharp, like "needle pricks", with occasional radiation up to the elbow. Once or twice a month he felt as though "bugs were crawling underneath his skin" over left wrist. In the past, he had tried gabapentin, amytriptyline, propoxyphene, acetaminophen with codeine and physical therapy without benefit. He reported some pain relief with topiramate and tramadol. He was not able to continue these medications due to financial reasons, except acetaminophen with hydrocodone. On examination, there were no clinical features of complex regional pain syndromehe did not meet the physical criteria for CRPS except for non-dermatomal pain and hyperalgesia over the wrist. His imaging studies where unremarkable. For diagnostic purposes, a In order to determine if the pain is sympathetically mediated, a local anesthetic left stellate ganglion block was performed, after informed consent, under ultrasound guidance with immediate relief of pain and complete pain relief for over five weeks. Having established the pain as responsive to stellate ganglion block, we elected to repeat the block under ultrasound guidance with steroid. Following informed consent and establishment of peripheral intravenous access, standard ASA monitors were applied. A preliminary scout scan including color flow Doppler was performed. Pain medicine fellow, experienced in performing ultrasound guided blocks, performed the procedure. With the patient supine on the procedure table, the left side of the neck was prepped with betadine and sterile drapes applied. A skin wheal was raised with 1% lidocaine over the posterolateral part of the transducer for an in-plane approach. Under ultrasound guidance, a 3.5 inch, 25 G needle was advanced to approximately 2-3 cms during attempts to visualize the needle tip (Figure 1). The patient experienced severe paraesthesia in the arm which necessitated immediate removal of the needle. Following recovery from the paraesthesia, in approximately five minutes, the needle was reintroduced at a different angle. Only portions of the needle were visualised along with tissue movement. As the needle tip could not be visualized clearly, 1 ml of injectate from a 10ml mixture containing equal volumes of 1% lidocaine, 0.25% bupivacaine and 40mg of Depomedrol was injected after a color flow Doppler exam and negative aspiration for blood. Immediately following the injection, the patient experienced severe ringing in both ears. Aspiration at this time was positive for blood. The needle was completely withdrawn and preparations made for resuscitation anticipating convulsions. Oxygen was administered via face mask while his vital signs continued to be monitored. Gradually, over 15 minutes, ringing in his ears decreased. He did not develop any other symptoms or signs of local anesthetic toxicity. At the patient's request, as he had good pain relief with the prior left stellate ganglion block, the attending physician changed to an out of plane approach to avoid the brachial plexus and completed the ultrasound guided stellate ganglion block, without any further complications. A repeat test dose was administered and visualized real time under ultrasonography. Following the test dose, 10ml of a mixture of 1% Lidocaine and 0.25% Bupivacaine with 40mg of Depomedrol was injected, in small increments. Vital signs remained stable. There was an approximately 20C increase in temperature compared to the right hand. His pain in the wrist also decreased dramatically. At the conclusion of the procedure, patient was noted to have features of Horner’s syndrome including a left sided ptosis, without hoarseness of voice or swallowing difficulty. He was then discharged home after monitoring for 30 minutes post procedure. Two days post procedure, patient reported a mild discomfort in his throat and chest. He declined our recommendation to go to the Emergency room for evaluation. During his follow up visit in two months, he reported occasional feelings of fullness in his ears and tinnitus which also resolved over the next few months. He declined further interventions and elected to manage his pain conservatively with oxycodone 3 times daily.

## 3. Discussion

This case report of transient neuronal injury followed by vascular injury and intravascular injection in the same patient highlights the importance of trajectory planning and vigilance during ultrasound guided interventions. The incidence of neuronal injuries following ultrasound guided regional anesthesia has been reported to be around 0.04% (11). There are no similar reports for ultrasound guided pain interventions. Only until symptoms of paresthesia were reported by the patient, was neuronal contact evident secondary to poor needle tip visualization. The transient neuronal injury could have been avoided, if the trajectory planning had included the possibility of contacting the brachial plexus with the in-plane approach. Needle redirection and avoidance of injection subsequent to patient report of paresthesia, probably, averted a more severe or permanent nerve injury. Operator preferences, of in plane and out of plane approaches, may have less relevance in avoiding injuries than a thorough knowledge of anatomy and proper trajectory planning. Needle visualization during the performance of ultrasound guided interventions has always been a challenge. No clear advantage exists with any particular needle (13). Smaller gauge needles, as used in this report, are more challenging to visualize. Validity of scout scanning and hydrolocalization techniques in regional anesthesia was starting to get acknowledged as "standard of care" at the time when these complications occurred (10, 14). Intravascular injection of local anesthetic may result in neurological as well as cardiovascular symptoms and signs including, perioral numbness, tingling, tinnitus, confusion, dysarthria, convulsions, dysrhythmias, and hypotension. Aspiration for blood, prior to injection, is not always reliable (12). Hydrolocalization, during real time imaging with the use of normal saline or dextrose, may facilitate identification of accidental intravascular injections (14). Injuries to the vertebral or the inferior thyroidal arteries have been reported during stellate ganglion block and hence trajectory planning may also have to include the location of these vessels (15, 16). In addition, the use of particulate steroids in the neck with the potential for intravascular injection especially into the vertebral artery could have dire consequences including stroke and spinal cord injury. Retropharyngeal hematoma is a complication which can be potentially fatal secondary to airway compromise. Features of retropharyngeal hematoma include, pain in the head, neck and chest. Late features include hoarseness, stridor, dysphagia, superior mediastinal obstruction and tracheal deviation (3). The symptoms in some instances can be delayed for more than two hours (17). All reports of retropharyngeal hematoma, thus far, have resulted from land mark based techniques. As an immediate examination was not performed, upon report of throat and chest discomfort, we can only presume that he may have developed a small hematoma which subsequently resolved. Stellate ganglion block has been used to manage hearing loss as it produces an increase in cochlear blood flow (18). We could postulate that the persistence of tinnitus at two month follow up may be because of the persistent increase in cochlear blood flow secondary to stellate ganglion block. A question, which then remains unanswered, is why does not it occur in every successful stellate ganglion block? The only difference in this particular case report is the accidental injection of a mixture containing a small amount of particulate steroid. Did the intravascular injection of particulate steroid alter the endolymphatics or was the local concentration high enough to cause damage to the hair cells? These questions may remain unanswered until further studies are performed. The primary reasons for the complications in this report are improper trajectory planning, inadequate needle tip visualization and injecting local anaesthetic with steroid for real time visualization of injectate. It is possible that these complications may have been avoided, if, besides a thorough assessment during the scout scanning, a larger size needle, hydrolocalization with saline or water, echogenic needle or similar needle visualization options were utilized. This is a report of a combination of neurological and vascular complications that occurred during the performance of an ultrasound guided stellate ganglion block. This report increases awareness about safer techniques for the performance of ultrasound guided pain interventions.
